# Clinical and molecular implications of mosaicism in *FMR1* full mutations

**DOI:** 10.3389/fgene.2014.00318

**Published:** 2014-09-17

**Authors:** Dalyir Pretto, Carolyn M. Yrigollen, Hiu-Tung Tang, John Williamson, Glenda Espinal, Chris K. Iwahashi, Blythe Durbin-Johnson, Randi J. Hagerman, Paul J. Hagerman, Flora Tassone

**Affiliations:** ^1^Department of Biochemistry and Molecular Medicine, School of Medicine, University of California at DavisDavis, CA, USA; ^2^Department of Public Health Sciences, School of Medicine, University of California at DavisDavis, CA, USA; ^3^Department of Pediatrics, School of Medicine, University of California at DavisDavis, CA, USA; ^4^MIND Institute, UC Davis Medical CenterSacramento, CA, USA

**Keywords:** mosaicism, fragile X, *FMR1*, FMRP, methylation, premutation

## Abstract

Expansions of more than 200 CGG repeats (full mutation) in the *FMR1* gene give rise to fragile X syndrome (FXS) through a process that generally involves hypermethylation of the *FMR1* promoter region and gene silencing, resulting in absence of expression of the encoded protein, FMRP. However, mosaicism with alleles differing in size and extent of methylation often exist within or between tissues of individuals with FXS. In the current work, CGG-repeat lengths and methylation status were assessed for eighteen individuals with FXS, including 13 mosaics, for which peripheral blood cells (PBMCs) and primary fibroblast cells were available. Our results show that for both PBMCs and fibroblasts, *FMR1* mRNA and FMRP expression are directly correlated with the percent of methylation of the *FMR1* allele. In addition, Full Scale IQ scores were inversely correlated with the percent methylation and positively correlated with higher FMRP expression. These latter results point toward a positive impact on cognition for full mutation mosaics with lower methylation compared to individuals with fully methylated, full mutation alleles. However, we did not observe a significant reduction in the number of seizures, nor in the severity of hyperactivity or autism spectrum disorder, among individuals with mosaic genotypes in the presentation of FXS. These observations suggest that low, but non-zero expression of FMRP may be sufficient to positively impact cognitive function in individuals with FXS, with methylation mosaicism (lowered methylation fraction) contributing to a more positive clinical outcome.

## Introduction

Fragile X syndrome (FXS) is the most common heritable form of intellectual disability and is caused by an expansion of a CGG trinucleotide repeat tract in the 5' UTR of the *FMR1* gene on the X chromosome. CGG-repeat expansion to greater than 200 repeats generally leads to DNA methylation(Sutcliffe et al., 1992; Alisch et al., 2013), aberrant heterochromatinization (Coffee et al., 2002; Tabolacci et al., 2008), subsequent silencing of the *FMR1* gene (El-Osta, 2002), and consequent loss of the corresponding gene product, FMRP (Pieretti et al., 1991; Godler et al., 2010). FMRP is an RNA binding protein that functions as a translational repressor at synapses (Antar and Bassell, 2003; Bagni and Oostra, 2013; Darnell and Klann, 2013; Sidorov et al., 2013). FMRP is important for learning and memory, and its absence is associated with the characteristic features of FXS, including intellectual disability, cognitive impairments and behavioral problems, autism spectrum disorders (ASD), Attention Deficit Hyperactivity Disorder (ADHD), seizure, in addition to hyper-responsiveness to sensory stimuli, hyperactivity, impulsive behavior, gaze aversion and shyness (Hull and Hagerman, 1993; Hagerman, 2002; Smith et al., 2012; Schneider et al., 2013; Ballinger et al., 2014; Machalicek et al., 2014; Maurin et al., 2014; Thurman et al., 2014).

Since the mapping of the *FMR1* gene to the X chromosome in 1991 (Verkerk et al., 1991), reports have emerged on mosaicism in both the *FMR1* allele size and methylation status within lymphocytes as well as between tissue types (Tarleton et al., 1992; Willems et al., 1992; Wohrle et al., 1992; Hagerman et al., 1994; Nolin et al., 1994; Dobkin et al., 1996; Taylor et al., 1999; Tassone et al., 2000a; Han et al., 2006; Govaerts et al., 2007; Hantash et al., 2010; Pretto et al., 2014). The CGG repeats within the *FMR1* gene predispose it to instability and the occurrence of size mosaicism, in which individuals present with different CGG repeat allele sizes such that some cells carry a full mutation allele while others carry a premutation allele; a situation that is common among individuals with FXS (Loesch et al., 2004; Lokanga et al., 2013). Notably, Nolin et al. (1994) analyzed a group of affected males with FXS by Southern Blotting and found 41% to be size mosaic. In addition to “size” mosaicism, some individuals exhibit “methylation” mosaicism, in which some cells have fully methylated full mutation alleles while other cells possess unmethylated full mutation alleles (Nolin et al., 1994; Tassone et al., 1999b; Genc et al., 2000). Methylation of the full mutation CGG repeats occurs early in embryonic development and may play a role in stabilization of the expanded repeats (Devys et al., 1992; Malter et al., 1997; Eiges et al., 2007). The hypermethylated state of the fragile X full mutation is found associated locally with histone deacetylation and chromatin remodeling (Coffee et al., 1999, 2002; El-Osta, 2002; Alpatov et al., 2014), and with transcriptional silencing of the gene (Pieretti et al., 1991; Pietrobono et al., 2005; Tabolacci et al., 2008).

Unmethylated alleles from the premutation to full mutation range are virtually transcriptionally active; specifically 2–10 fold overexpressed (Tassone et al., 2000a; Kenneson et al., 2001; Peprah et al., 2010). Overexpression of expanded CGG-repeat alleles is currently believed to lead to “toxicity” of the *FMR1* mRNA (Hagerman, 2013; Pretto et al., 2014). However, because expanded CGG-repeat alleles are translated with reduced efficiency (Kenneson et al., 2001; Primerano et al., 2002; Brouwer et al., 2007; Ludwig et al., 2011) FMRP expression is negatively correlated with the CGG repeat number particularly in the upper premutation and full size range (Ludwig et al., 2014; Pretto et al., 2014). Thus, *FMR1* and FMRP expression in a mosaic background could add complexity to the clinical presentation and widen the spectrum of involvement in FXS mosaics. This expectation stems from observations of inter- and intra-tissue *FMR1* methylation and size mosaicism in premutation carriers and a correlation between methylation and the number of clinical symptoms in a group of premutation carriers with alleles partially methylated (Allingham-Hawkins et al., 1996; Tassone et al., 1999b; Pretto et al., 2014). In addition, while somatic size or methylation mosaicism is not considered as part of a prognostic evaluation, several reports suggest that mosaicism can impact the penetrance of the disorder and that the methylation status of full mutation mosaics affects cognitive functioning (McConkie-Rosell et al., 1993; Hagerman et al., 1994; Schmucker et al., 1996; Wohrle et al., 1998; Helderman-van den Enden et al., 1999). Specifically, McConkie-Rosell et al. (1993), studied a fragile X family with 6 brothers and found that the degree of phenotypic expression of FXS correlates with the degree of *FMR1* methylation. The effect of the methylation status was likely due to the overall FMRP levels, since there is a correlation between methylation status and FMRP production (de Vries et al., 1996; Tassone et al., 1999b). A large study of 318 families (2253 individuals) reported that 12% of full mutation males and 6% of females exhibited mosaicism (Rousseau et al., 1994). Notably, a study of 46 males with FXS under 20 years of age was assessed for development of communication, self-care, socialization and motor skills as a function of presence or absence of mosaicism. They reported that adaptive skills development was 2–4 times greater in mosaic cases than in cases with a full mutation suggesting that phenotypic severity can be influenced by the presence of mosaicism (Cohen et al., 1996).

Additional cases of methylation mosaicism have also been reported and support the notion that cognitive function negatively correlate with both the length of CGG repeats and the methylation status (Merenstein et al., 1994; Mueller et al., 1995; Smeets et al., 1995; Schmucker et al., 1996; Wohrle et al., 1998; Helderman-van den Enden et al., 1999; Han et al., 2006; Loesch et al., 2012). Methylation mosaicism has also been associated with higher IQ scores and lower phenotypic presentation in post-pubescent males when compared to males with fully methylated full mutations (Merenstein et al., 1996) and a direct correlation between methylation status and FMRP levels has been demonstrated before (de Vries et al., 1996; Tassone et al., 1999a; Godler et al., 2010; Loesch et al., 2012; Pretto et al., 2014).

In this study we have investigated the expression of *FMR1* and FMRP in peripheral blood mononuclear cells (PBMCs) and fibroblast cells derived from *FMR1* full mutation and mosaic subjects. We report on inter- and intra-tissue mosaicism in eighteen individuals with FXS, 13 of whom were methylation and/or size mosaics, for which peripheral blood cells (PBMCs) and primary fibroblast cells were available. Using simple linear regression analysis we also investigated the genotype/phenotype relationship including clinical measures such as IQ, seizures, ASD, and ADHD.

## Materials and methods

### Subjects

Individuals were recruited through the MIND Institute Fragile X Research and Treatment Center. Participants provided informed consent according to protocols approved by the UC Davis Institutional Review Board. Eighteen participants with the FXS mutation, belonging to three mutational categories, were included in this study: fully methylated, full mutation males (*n* = 2) and females (*n* = 3); males (*n* = 12) and females (*n* = 1) with methylation and size mosaicism. Individuals who were size mosaics and/or methylation mosaics were combined for the purpose of molecular analysis in a mosaic group. Ages ranged from 13 to 73 years (mean ± *SD* = 31 ± 18 years).

### Clinical measures

Clinical assessment of participants included the domains of FSIQ, ASD, ADHD, perseveration, tantrums, anxiety, and seizures. Cognitive testing was carried out with standardized IQ measures as indicated in Table 1 (Mullen and American Guidance Service, 1995; Wechsler, 1997, 2009; Psychological Corporation, 2002; Wechsler et al., 2008); ASD was assessed with the Autism Diagnostic Observation Scale (ADOS) using a module that was developmentally appropriate (Derogatis, 1994; Lord, 2002); ADHD was determined by clinical assessment (Swanson et al., 2001) along with a history of perseverations, tantrums and anxiety (Derogatis, 1994).

**Table 1:** **Table shows demographic information, mutation category, and molecular outcome measures for the cases included in this study**.

**Case**	**Gender**	**Age**	**Category**	**IQ**	**IQ test**	**# Clinical measures**	**PBMCs**					**Fibroblasts**				
							**CGG size**	**% Meth**	**AR**	***FMR1* mRNA (Std. Err)**	**FMRP**	**CGG size**	**% Meth**	**AR**	***FMR1* mRNA (Std. Err)**	**FMRP**
1	F	13	FM	72	Binet	3	29, 570, 710, 1050		0.68	0.61 (0.10)	0.031	29, 570, 710, 1050		0.3	0.16 (0.01)	0.025
2	F	15	FM	83	SB-5	N/A	30, 450, 650		0.82	1.06 (0.09)	0.080	30, 450, 650		0.52	0.30 (0.01)	0.300
3	F	61	FM	69	SB-5	N/A	31, 430, 670		0.85	1.86 (0.08)	0.075	21, 680		0.4	0.28 (0.01)	0.645
4	F	26	FM/ MM	80	WISC	1	27, 320–850 (UM smear)	>95	0.46	5.75 (0.01)	N/A	27, 720, 940, 1060, 1400 (140)	54	0.55	N/A	N/A
5	M	30	FM	55	WAIS-III	5	370	100		0.04 (0.00)	0.0	370	100		0.002 (0.00)	0.010
6	M	26	FM/ MM	51	WAIS-III	5	270, 390, 720 (UM smear)	>95		0.20 (0.01)	0.002	290, 540, 790 (UM smear)	>95		0.06 (0.01)	0.040
7	M	26	FM	54	SB-5	N/A	490, 680, 960	100		0.03 (0.01)	0.0	500, 820, 960	100		0.00	0.00
8	M	32	FM/ SM	63	WAIS-III	N/A	510, 590, 670, 810 (66)	76		0.45 (0.09)	0.027	510, 590, 670, 810	100		N/A	N/A
9	M	61	FM/ MM	64	WAIS-III	1	250 (UM smear)	>95		1.24 (0.08)	0.003	250 (UM smear)	>95		0.29 (1.00)	0.070
10	M	73	FM/ SM	40	SB-5	N/A	290 (80–245)	40		2.03 (0.06)	0.024	290 (200–250)	78		0.28 (0.02)	0.141
11	M	32	FM/ MM	45	Weschler	N/A	610 (260–450)	74		0.39 (0.03)	0.003	425, 580, 680 (210–280)	79		0.58 (0.06)	0.199
12	M	25	FM/ MM	55	WASI	2	345, 450, 650 (215)	68		1.97 (0.08)	0.009	235, 350, 650 (UM smear)	82		0.17 (0.02)	0.067
13	M	16	FM/ SM	73	WPPSI-III	4	500, 820, 980, 1100 (150–230)	90		1.12 (0.28)	N/A	390, 490, 950, 1140 (160–230)	94		0.16 (0.01)	0.147
14	M	58	FM/ SM	64	WAIS-III	3	180 (30–260)	37		2.19 (0.01)	0.018	200 (250–760)	21		0.29 (0.06)	0.224
15	M	15	FM/ SM	63	SB	5	230 (30–570)	7		2.93 (0.11)	0.025	255 (260–380)	12		1.83 (0.22)	0.404
16	M	16	FM/ SM	72	SB	3	230, 330 (30–270)	14		2.57 (0.14)	0.010	230, 330 (30–200)	18		0.96 (0.02)	0.394
17	M	25	FM/ SM	79	WAIS-III	4	440 (65)	<5		2.29 (0.08)	0.075	190, 220, 400, 500, 800 (65)	49		0.77 (0.04)	0.453
18	M	29	FM/ MM	117	WAIS-III	3	180 (210–420)	7		3.85 (0.43)	0.019	180 (200–270)	16		1.32 (0.02)	0.474

*FM, full mutation; FM/MM, full mutation methylation mosaic; FM/SM, full mutation size mosaic; UM, unmethylated; N/A, non available*.

### PBMC isolation

Whole blood was collected in Cell Preparation Tube (CPT) vacutainers with sodium citrate (Becton Dickinson) and centrifuged according the manufacturer's recommendations to separate mononuclear cells from whole blood. PBMCs were washed with Dulbecco's phosphate buffered saline (PBS) and frozen in RPMI 1640 media with 10% fetal bovine serum and 10% dimethyl sulfoxide.

### Fibroblast cell lines

Explants of ~3-mm dermal biopsies were minced and placed in a 100-mm TC-treated tissue culture dish (Corning Life Science, USA) with a small drop of Fibroblast medium [Gibco AmnioMAX-C100 Basal Medium supplemented with 15% AmnioMAX-C100 Supplement (Invitrogen, Carlsbad, CA, USA). Dishes were incubated at 37°C in a humidified 5% CO_2_ atmosphere and media was replaced every 3–4 days. Fibroblast outgrowths were harvested by trypsinization, transferred into a new dish with a modified Fibroblast Medium (1:1 solution of Gibco AmnioMAX-C100 supplemented with 15% AmnioMAX-C100 Supplement (Invitrogen, Carlsbad, CA, USA) and RPMI-1640 Basal Medium supplemented with 10% fetal bovine serum (Invitrogen), 1X Primocin (Invitrogen), 1% non-essential amino acids (Invitrogen), with media exchange every 3–4 days and allowed to reach 90% confluence prior to splitting. Fibroblast cultures were passaged no more than 3 times prior to collection for DNA, RNA, or protein extracts isolation or cryopreservation.

### CGG repeat length

DNA from PBMCs or primary fibroblast cell lines was isolated using Gentra Puregene Blood Kit (Qiagen, Valencia, CA). DNA was analyzed by Southern Blot as previously described to establish CGG repeat length, percentage of CpG methylated *FMR1* alleles, and, in females, activation ratio was also determined (Tassone et al., 1999a, 2008). CGG repeat length was also measured by PCR amplification followed by capillary electrophoresis, as previously described (Chen et al., 2010; Filipovic-Sadic et al., 2010).

### FMR1 mRNA expression levels

Whole blood was collected in Tempus vacutainers (Applied Biosystems, Foster City, CA) and processed to isolate total RNA. Fibroblast cell total RNA was isolated using Trizol (Qiagen, Valencia, CA). Total RNA was reverse transcribed into cDNA, quantitative real time PCR (qRT-PCR) was performed on cDNA template to determine *FMR1* mRNA levels. *FMR1* mRNA levels were reported in relative abundance compared to reference gene β-glucoronidase (*GUS*). Details of the qRT-PCR methodology are as previously described (Tassone et al., 2000b).

### FMRP expression levels by western blot analysis

PBMCs and fibroblast cells were thawed on ice and gently pelleted at 5000 rpm for 5 min. Cell pellets were lysed with rapid shaking at 70°C using a thermomixer (Eppendorf) for 20 min. Lysis buffer contained 0.125 M Tris HCL (pH 6.8), 2% SDS, 10% Glycerol, and 5% BME. The samples were cooled to room temperature and centrifuged at 13,000 rpm in a benchtop centrifuge for 10 min after which the protein extracts were transferred to clean microcentrifuge tubes. Protein concentrations were measured using the Detergent Compatible Protein Assay RC/DC (Bio-Rad Laboratories Inc., United States). Proteins (10 μg) were separated by electrophoresis in Any KD Criterion TGX Gels (Bio-Rad Laboratories Inc., United States) in 1X Tris/Glycine/SDS buffer and transferred overnight at 4°C, 30 volts, to 0.2 μm nitrocellulose membranes (Bio-Rad Laboratories Inc., Germany) in 1X Tris/Glycine/SDS buffer containing 10% methanol. The membranes were blocked using Licor blocking buffer (Licor) with 50% 1X PBS for 1 h and hybridized overnight at 4°C with 1:10,000 chicken anti-GAPDH (Millipore) and 1:5000 mouse anti-FMRP (Chemicon, Millipore). Membranes were washed with 1X PBST and hybridized with secondary antibodies for 1 h at room temperature (1:25,000 goat anti-chicken IRDye 800CW and 1:20,000 goat anti-mouse IRDye 680LT; Licor). Membranes were washed in 1X PBST and finally 1X PBS prior to being imaged using Licor Odyssey System with Image Studio Version 2.1 (Licor).

### Statistical analysis

*FMR1* expression was compared between groups, adjusting for percent methylation, using a multiple regression model including group and percent methylation. Percent methylation values indicating >95% were converted to 95% for use in analysis. FMRP expression was compared between groups using one-way analysis of variance. *FMR1* expression, FMRP expression, IQ, numbers of clinical features, and percent methylation were correlated with each other using simple linear regression. FMRP expression was correlated with *FMR1* expression, adjusting for percent methylation, using multiple linear regression analysis. *FMR1* data and FMRP expression were log-transformed prior to analysis whenever they were used as the response variable in a linear model. Analyses were conducted using R, version 3.0.3 (Team, 2014).

## Results

### Clinical history of two representative cases

The group of patients examined in this study consists of 18 subjects that presented with clinical characteristics of FXS and molecularly with size or methylation mosaicism. The clinical history of two of the 18 cases is described in detail below.

**Figure 1:**
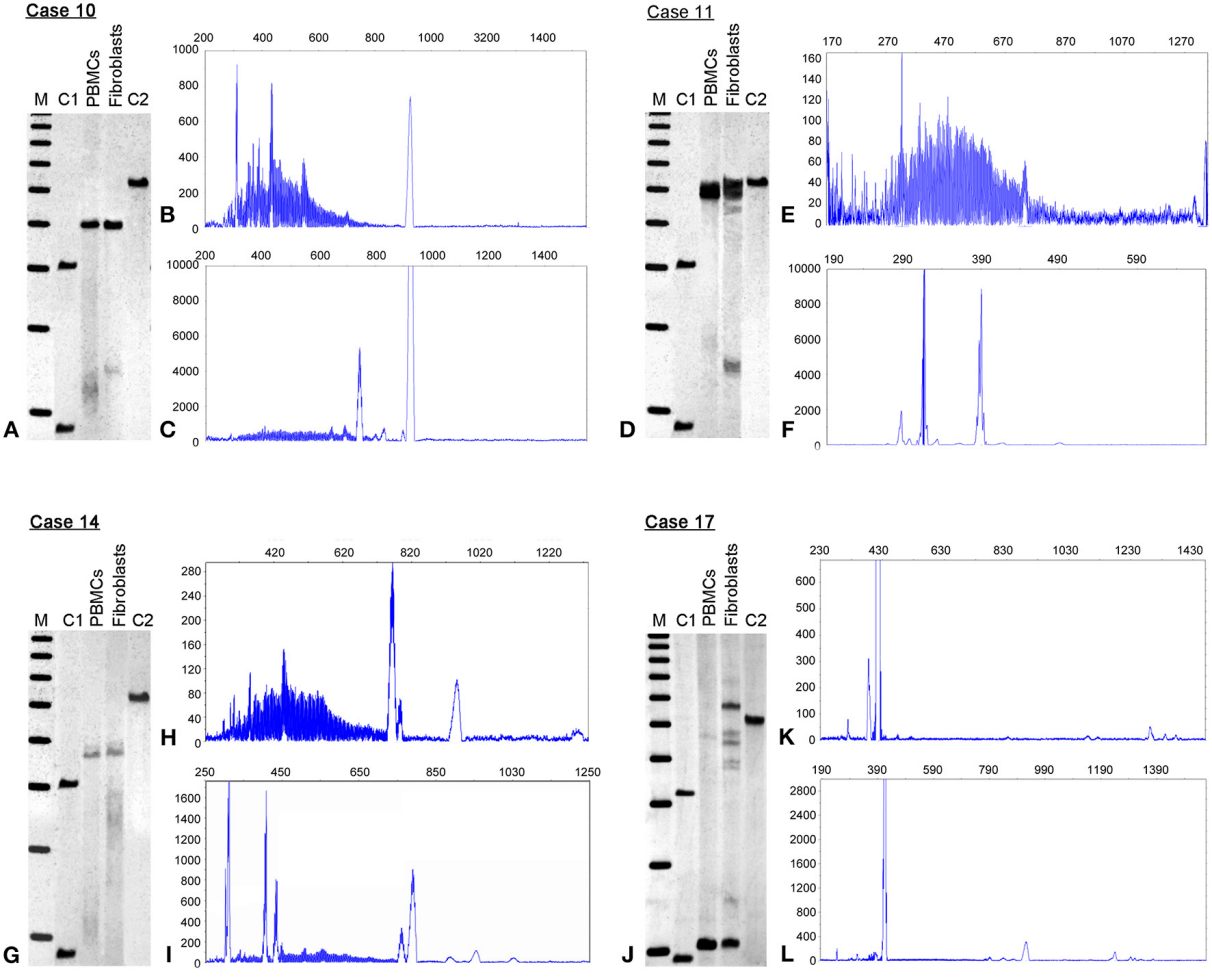
**Methylation status and CGG size instability**. Southern blots (SB) and electrophoregrams in four representative cases of full mutation mosaic males, cases 10, 11, 14 and 17 are illustrated. The SB analysis (left, **A,D,G,J**) demonstrates differences in *FMR1* percent of methylation when comparing peripheral blood lymphocytes (PBMCs) to primary cultured fibroblasts (Fibroblasts). See also Table 1. M = 1 Kb DNA size ladder marker; C1, normal female, negative control and C2, full mutation male, positive control. The electrophoregrams show CGG instability as illustrated by the presence of serial peaks, each representing single distinct alleles, with differences between PBMCs **(B,E,H,K)** and fibroblasts **(C,F,I,L)**. The X axis marks the size of the alleles in base pairs. The Y-axis marks the fluorescence intensity of each allele.

**Case 14** is a 58 year old male with a history of learning problems and shyness in childhood. He was dyslexic, and had difficulty reading and writing. On evaluation at age 55, he demonstrated psychotic symptoms on the Structured Clinical Interview for DSM IV (SCID) with both visual and auditory delusions. He was also subthreshold for agorophobia. He has developed a neuropathy in the last year with pain, numbness, and tingling in both his hands and in his feet, with burning pain in his feet particularly evident during the past few months. He trips frequently, but has not reported ataxia or tremor by history or by examination. His family history includes a mother who was a premutation carrier with dementia, tremor, and ataxia. His brother has FXTAS, his sister has FXS, and his daughter is a premutation carrier with a son affected with FXS. On molecular testing, he displays substantial somatic instability (size mosaicism) as evidenced by a broad range of CGG repeat alleles more so in PBMCs compared to fibroblasts (Figures 1H,I). He also has methylation mosaicism in both PBMCs and fibroblasts (Figure 1G) with most of the cells carrying unmethylated alleles in both PBMCs (63%) and fibroblasts (79%), although the CGG repeat sizes were larger in the fibroblasts (Table 1). *FMR1* mRNA levels were over two-fold higher than normal (2.19 ± 0.01; mean *FMR1* mRNA in controls = 1.42 ± 0.26; Tassone et al., 2000b) in PBMCs, and but slightly lower compared to normals in fibroblasts (0.29 ± 0.06; mean in *FMR1* mRNA in controls = 0.40 ± 0.08; Garcia-Arocena, 2010). His FMRP expression levels were low; approximately 10% of normal in PBMCs and in fibroblasts, likely due to inefficient translation of large expanded alleles.

**Case 17** presented at 20 years of age with a history of a normal pregnancy; but with delayed development, hypotonia, ADHD, shyness, and social anxiety during early childhood. He also had seizures documented by EEG during his first 2 years, and night terrors during his first 5 years. He has characteristic FXS behaviors including intermittent poor eye contact, tactile defensiveness, social anxiety, sensitivity to stimuli, and perseveration. However, he does not have tantrums or aggression. He has had a joint dislocation of his toe, and right exotropia. He has a large head circumference (>99%) with mild obesity. His ears are large and prominent with cupping bilaterally, his fingers are hyperextensible and his skin is soft and velvet-like, although striae are present over his lateral chest and abdomen. He has flat feet with a moderate degree of pronation. He has a high arched palate, and macroorchidism with a testicular volume of 50 mL bilaterally. He was able to tandem walk without difficulty, which is unusual for a man with FXS. He is higher functioning than most adult males with FXS since his FSIQ is in the borderline to low normal range: WAIS-III FSIQ is 79; performance IQ 78; and verbal IQ is 83, with a verbal comprehension score of 93, perceptional organization of 70, working memory of 69, and perceptional speed of 79. At the molecular level, he showed size mosaicism in PBMCs and fibroblasts with a premutation allele of 65 CGG repeats and full mutation alleles present in a small percent of PBMC cells (<5%), but much higher than in fibroblast cells (49%) (Figures 1J,K; Table 1). *FMR1* mRNA levels were over 2-fold higher than normal both in PBMCs and in fibroblasts (Table 1). Within the mosaic group, he showed a higher FMRP expression in both tissues likely due to the premutation allele of 65 CGG repeat present in over 50% of the cells (Table 1).

### Differences in methylation and size mosaicism occur between PBMCs and fibroblasts

Southern Blot and PCR analyses were used to determine the methylation status and the CGG repeat size of the *FMR1* allele in both PBMC and fibroblast samples from individuals with FXS (males, *n* = 14 and females, *n* = 4) (Table 1). Among the FXS cases, three females had both a normal allele and a full mutation allele in both tissues (Cases 1–3, Table 1) while one female had size and methylation mosaicism that differed between PBMCs and fibroblasts (Case 4, Table 1). Two males with FXS showed fully methylated full mutation alleles (Cases 5 and 7), while the remaining 12 (Table 1) were methylation or size mosaics in both PBMCs and/or fibroblasts. The percent of methylation and the CGG size varied between PBMCs and fibroblast cells in the majority of cases (Table 1). Variations in CGG size and methylation status, as observed by Southern Blot and PCR assay, for 4 representative cases are shown in Figure 1.

In the methylation mosaic males, the CGG size of the unmethylated alleles spanned the full mutation range in all individuals; in some cases alleles within the premutation and normal range were present, particularly in the PBMCs (Table 1). However, it was not possible to estimate the molecular contribution and the consequent potential clinical outcome due to any single allele present in the sample. Although the observed differences in methylation were variable between individuals and involved both differences in percent of methylation and allele size, the percent of methylation was consistently higher in the majority of the fibroblast cells. The activation ratio, expressing the percent of cells carrying the normal allele on the active X chromosome, was higher in PBMCs than fibroblast cells for three of the four female cases (Cases 1–3) while the remaining female (Case 4) had approximately the same activation ratio between cell types (0.46 in PBMCs, 0.55 in fibroblast cells) (Supplementary Figure 1). The higher activation ratio in Cases 1–3, and thus the higher proportion of normal alleles on the active X chromosome, likely reflects the *FMR1* mRNA levels observed in the PBMCs of these individuals closer to the levels observed in normal controls (mean *FMR1*mRNA in controls = 1.42 ± 0.26; Tassone et al., 2000b). However, the FMRP levels were less than 50% of the control levels for both PBMCs and fibroblasts (mean FMRP in control PBMCs = 0.17 ± 0.06; mean FMRP in control fibroblasts = 1.2 ± 0.02; data not shown) (Table 1).

### FMR1 mRNA and FMRP expression levels are higher in individuals with mosaicism

**Figure 2:**
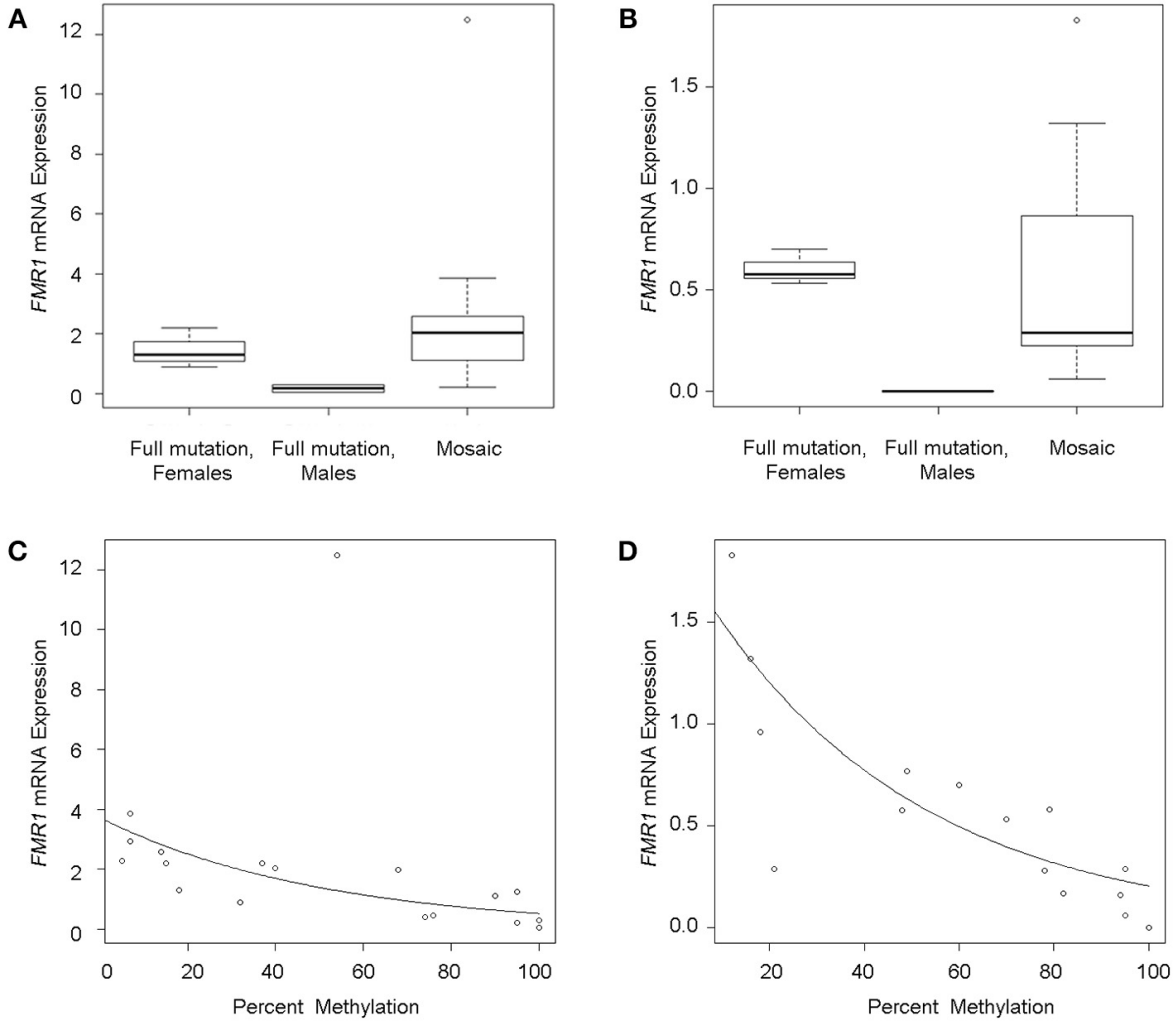
***FMR1* gene expression**. Boxplots show *FMR1* expression in PBMCs **(A)** and fibroblasts **(B)** for full mutation females, full mutation males and mosaic individuals as measured by qRT-PCR relative to a reference gene (*Gus)*. The line across each box represents the group median. Outliers are shown as circles. Scatterplots of *FMR1* mRNA expression levels (y-axis) as a function of percent methylation (x-axis) show single data points demonstrating decrease *FMR1* mRNA expression with increased methylation in PBMC's **(C)** and in fibroblasts **(D)**. Circles represent observed data. The solid line shows the linear regression fit.

**Figure 3:**
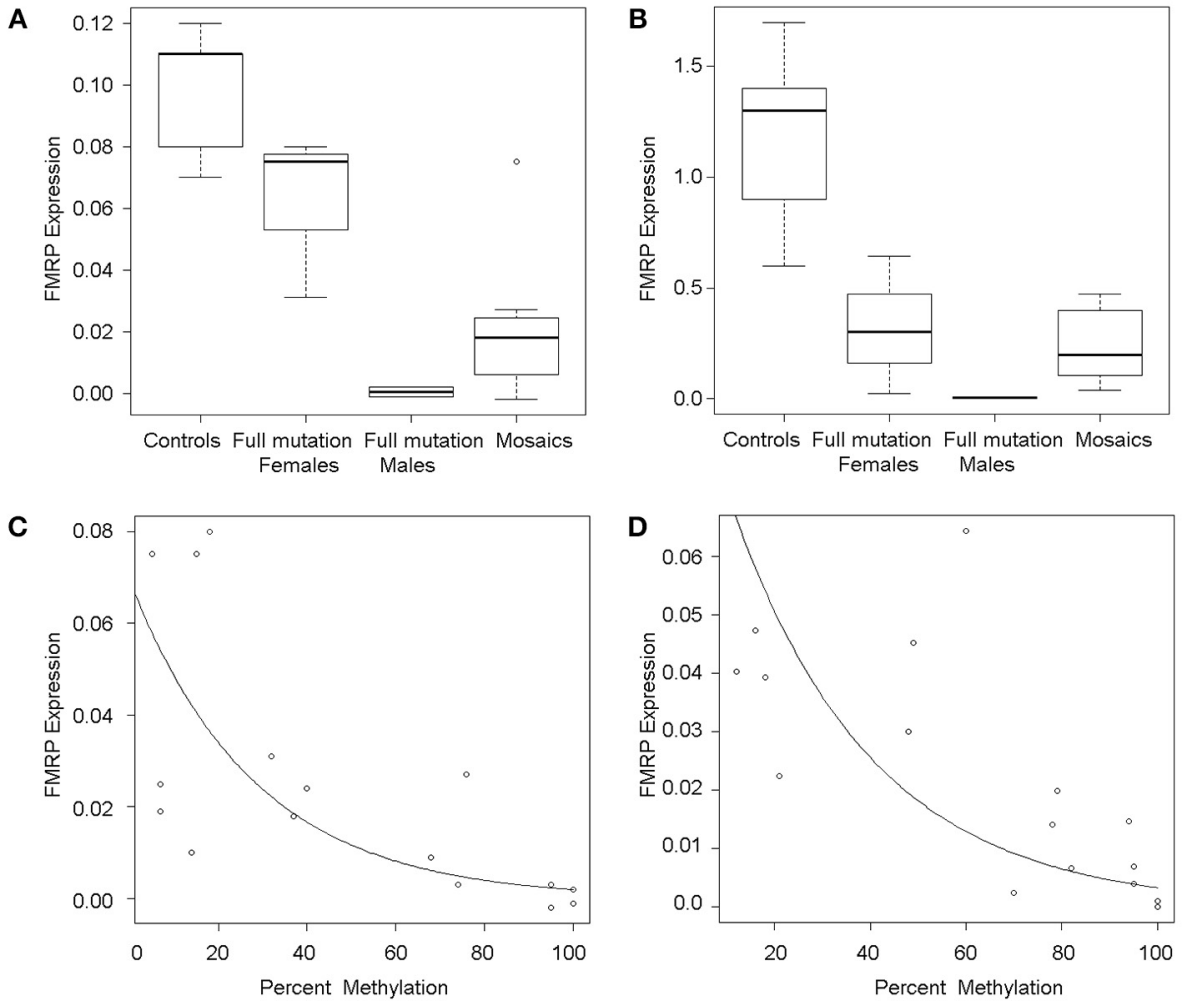
**FMRP expression**. Boxplots show semi-quantitative measures of FMRP expression in PBMCs **(A)** and fibroblasts **(B)** for control individuals, full mutation females, full mutation males, and mosaic individuals as detected by Western Blot analysis. The line across each box represents the group median and outliers are shown as circles. Scatterplots show single data points for FMRP expression (y-axis) as a function of percent of methylation in PBMCs **(C)** and fibroblasts **(D)**, illustrating an inverse association between FMRP levels and percent of methylation and in both cases, a dramatic loss of FMRP expression in highly methylated alleles.

*FMR1* mRNA levels adjusted for percent methylation were compared between the three mutational groups, namely males with full mutations, females with full mutations and individuals with mosaicism (methylation and size male and female mosaics, *n* = 13). *FMR1* mRNA levels measured by qRT-PCR varied between 0 and 5.75 (mean 1.79). As previously reported (Tassone et al., 2000a), *FMR1* mRNA levels were higher in both tissues in mosaics than in full mutation males (Table 1; Figures 2A,B). A correlation between *FMR1 mRNA* expression and percent of methylation in PBMCs (Figure 2C) and fibroblasts (Figure 2D) showed a decrease in *FMR1* expression with increased methylation using simple linear regression. FMRP expression between the three mutational categories showed a 5.1 fold-greater FMRP expression in the mosaic group compared to the full mutation males in PBMCs (Figure 3A) (although this was not statistically significant, *P* = 0.199) and significantly higher in the female group compared to full mutations (*p* < 0.001). FMRP was 37.0-fold greater in mosaic males and females than in full mutation males (*P* = 0.001) in fibroblast cells (Figure 3B). FMRP expression in PBMCs and fibroblasts decreased significantly with increasing percent methylation (*P* = 0.001 and *p* = 0.02 respectively; Figures 3C,D).

### FMRP expression levels correlate with IQ

**Figure 4:**
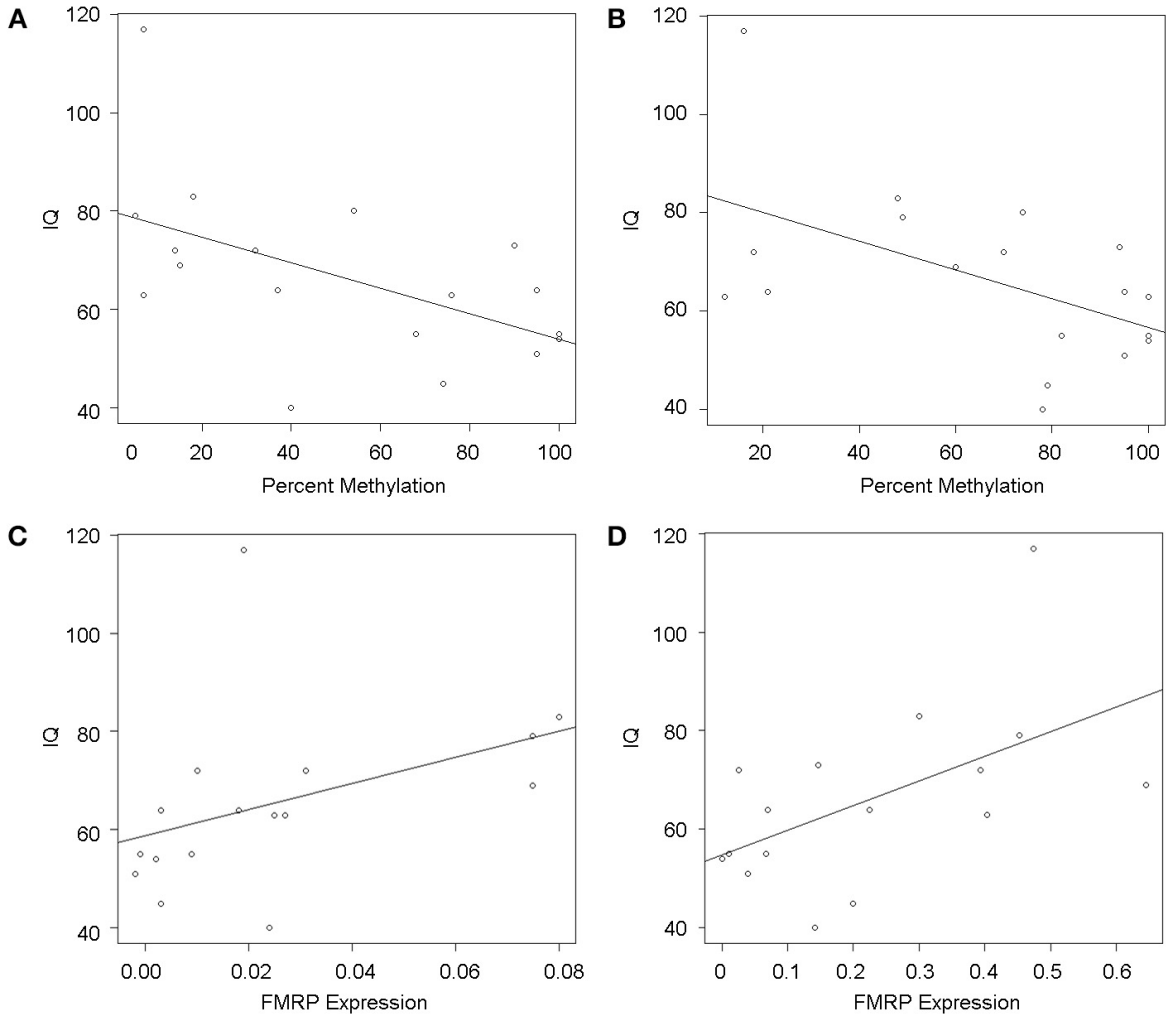
***FMR1* methylation and FMRP are associated with FSIQ scores as measured by Stanford Binet-5, WISC, WAIS-III, Weschler, and WPPSI-III Standardized tests**. Scatterplots show IQ scores (y-axis) as a function of percent methylation (x-axis) in PBMCs **(A)** and fibroblasts **(B)**, demonstrating a significant correlation between lower IQ and greater percent of methylation in both cell types. Circles represent observed data and the solid line shows the linear regression fit. Similarly, scatterplots of IQ scores (y-axis) as a function of FMRP expression show that lower IQs significantly correlate with reduced FMRP expression in fibroblasts **(D)** and a similar trend can be observed in PBMCs **(C)**. Circles represent observed data, the solid line shows the linear regression fit.

Participants FSIQ decreased significantly with increased *FMR1* methylation in both PBMCs (*P* = 0.022) and fibroblasts (*P* = 0.02) (Figures 4A,B) while the IQ increased significantly with increasing FMRP expression in fibroblasts (*P* = 0.028) (Figure 4D) while a similar trend, although not statistically significant, was observed in PBMCs (*P* = 0.118) (Figure 4C). Notably, the plots in Figures 4A,B show a wide scatter likely due to the large range of unmethylated CGG allele sizes, particularly in individuals with mosaicism (Table 1). Both CGG allele size and the methylation status affect the FMRP expression levels, which ultimately can influence the severity of the phenotype.

### Molecular measures do not correlate with the number of clinical features present

In order to determine whether the percent of methylation in *FMR1* correlated with clinical presentation we examined the relationship between methylation and number of clinical features that were diagnosed by their physician as reported in their medical records. Scores for severity of symptoms were not considered in our evaluation; only the presence or absence of each feature was taken into consideration. Clinical diagnoses used in this study included ASD, ADHD, perseveration, tantrums, anxiety, and seizures. A minimum of one (*n* = 2) and a maximum of five (*n* = 4) clinical features out of six considered were observed in the 18 participants for which this clinical information was available. Out of the 18 individuals with FXS, 50% (*n* = 9) presented with ASD. The number of clinical features was not significantly associated with percent methylation in either PMBCs (*P* = 0.926) or fibroblasts (*P* = 0.803). In addition, FMRP expression did not significantly correlate with the number of clinical features.

## Discussion

Reports of methylation and size mosaicism have been observed in a large proportion of individuals with a full mutation (McConkie-Rosell et al., 1993; Nolin et al., 1994; Genc et al., 2000; Tassone et al., 2000a; Loesch et al., 2012; Pretto et al., 2014). However, despite the fact that mosaics can produce some FMRP, depending on the methylation and the CGG allele size, they usually present with developmental delay. This is likely due to the limited amount of FMRP present, due both to lowered gene expression and the difficulty in translating residual mRNA with the expanded CGG repeat (Primerano et al., 2002; Ludwig et al., 2011, 2014). Alternatively, because DNA testing is normally performed in PBMCs, the results may not accurately reflect the mutation pattern in other tissues. Mosaicism in different tissues has been investigated and reported in several studies (Genc et al., 2000; Bonarrigo et al., 2014; Pretto et al., 2014), which have reported similarities across tissues in some cases and extreme difference in mosaicism in others. Thus, it is difficult to predict on an individual basis whether mosaicism observed in blood will reflect the pattern in other tissues, particularly brain, and therefore the patterns of clinical involvement/severity. However, the studies in general show association between mosaicism and prognosis (Merenstein et al., 1994; Mueller et al., 1995; Smeets et al., 1995; Dobkin et al., 1996; Schmucker et al., 1996; Wohrle et al., 1998; Helderman-van den Enden et al., 1999; Genc et al., 2000; Han et al., 2006; Govaerts et al., 2007; Loesch et al., 2012; Pretto et al., 2014).

In the current study the impact of mosaicism was assessed in 18 individuals with a full mutation by comparing molecular measures in both PBMC and fibroblast cells. In several cases CGG allele sizes and their corresponding *FMR1* and FMRP expression was similar between PBMC and fibroblast samples, however, noticeable differences in CGG size allele distribution were observed in the majority of cases (Table 1) likely contributing to the variations observed in the phenotypic presentation of FXS. Percent of methylation was in general lower in the PBMCs than in fibroblasts. Notably, the mRNA expression was higher in PBMCs suggesting that the unmethylated alleles even in the full mutation range are actively transcribed (Tassone et al., 2000). Although an excess in transcription, producing higher than normal levels of mRNA was observed for many of the subjects included in this study, these are long expanded alleles, which are inefficiently translated to FMRP (Primerano et al., 2002; Loesch et al., 2004; Peprah et al., 2010). Thus, it is not surprising that some individuals exhibited elevated *FMR1* mRNA but not lower FMRP levels.

The fully-methylated, full mutation males and those mosaics with a higher percent of cells carrying a methylated allele had an IQ rating under 70, within the range of intellectual disability, as is common in FXS. Higher IQ scores were observed in mosaics with less methylation (Table 1). This data suggest that low FMRP levels are biologically critical, and small increases in its expression may have a significant impact on cognitive function. Alternatively, it is also possible that different methylation and consequently different FMRP levels may exist in neural tissues. We found no significant correlation between percent of unmethylated alleles and the number of clinical phenotypes present, suggesting the importance of the genetic background, in addition to the presence of a full mutation in the complexity of the clinical phenotype.

As expected, the IQ was in the borderline range for females as the FXS phenotype in females is normally milder than that of FXS in males due the normal, active allele expressing FMRP at normal levels. The activation ratio, which reflects random X inactivation, was measured in the full mutation females in both PMBCs and fibroblasts, revealing inter-tissue difference that can have a biological and ultimately a clinical impact on a full mutation female (Table 1). Higher activation ratios are favorable, as they indicate more normal alleles being expressed. Because activation ratios vary between tissue types, measurements from PBMC or fibroblast samples may not reflect what may be present in clinically relevant tissue types such as brain.

In all cases the presence of full mutation alleles contributed to a lower IQ and perhaps to the higher incidence of ASD (50%) observed in these individuals.

Although mosaic individuals can have milder cognitive involvement, they can be at risk for developing FXTAS if their *FMR1* mRNA levels are elevated as suggested from cases previously reported in the literature (Loesch et al., 2012; Pretto et al., 2013, 2014; Santa Maria et al., 2013). In addition, individuals with both lowered FMRP and elevated *FMR1* mRNA can also be at greater risk for psychotic symptoms (Schneider et al., 2013).

One limitation of this study is represented by the small number of subjects (*n* = 18), particularly due to the wide range of CGG size and methylation, for which both PBMCs and primary fibroblasts were available. The results presented in this study, may have clinical relevance, as a detailed molecular diagnosis (including information about methylation status, *FMR1*mRNA and FMRP levels) could provide additional information and guide to clinicians and expectations for the family of a patient with FXS. However, it also underlines the complexity of the disorders with many molecular facets, including CGG repeat size, methylation, FMRP expression, and intra-tissue mosaicism that can lead to the broad spectrum of clinical involvement in FXS, particularly in those with mosaicism.

### Conflict of interest statement

Dr. Randi Hagerman has received funding from Roche, Novartis, Seaside Therapeutics, Forest Curemark and the National Fragile X Foundation for clinical trials in fragile X syndrome and/or autism. She has also consulted with Novartis, Genentech and Roche regarding treatment in fragile X syndrome. Dr. Flora Tassone has consulted with Novartis and Genentech and has received funds from Roche. The authors declare that the research was conducted in the absence of any commercial or financial relationships that could be construed as a potential conflict of interest.
