# Supplementary material for: Clinical and molecular implications of mosaicism in FMR1 full mutations
Source: Front Genet. 2014 Sep 17;5:318. doi: 10.3389/fgene.2014.00318 (PMC4166380; doi:10.3389/fgene.2014.00318)

Supplementary Figure 1. Southern blot analysis showing the comparison between PBMCs and fibroblasts illustrates difference in both methylation status and shows size instability in all four female cases. (M= 1kb marker, C1= negative control, and C2= positive control). The activation ratio, which indicates the percent of cells carrying the normal allele on the active X chromosome and measured by the intensity of the unmethylated normal allele (2.8Kb) over the sum of the unmethylated plus the methylated normal allele (5.2Kb), differs particularly in Case 1, 2 and 3.

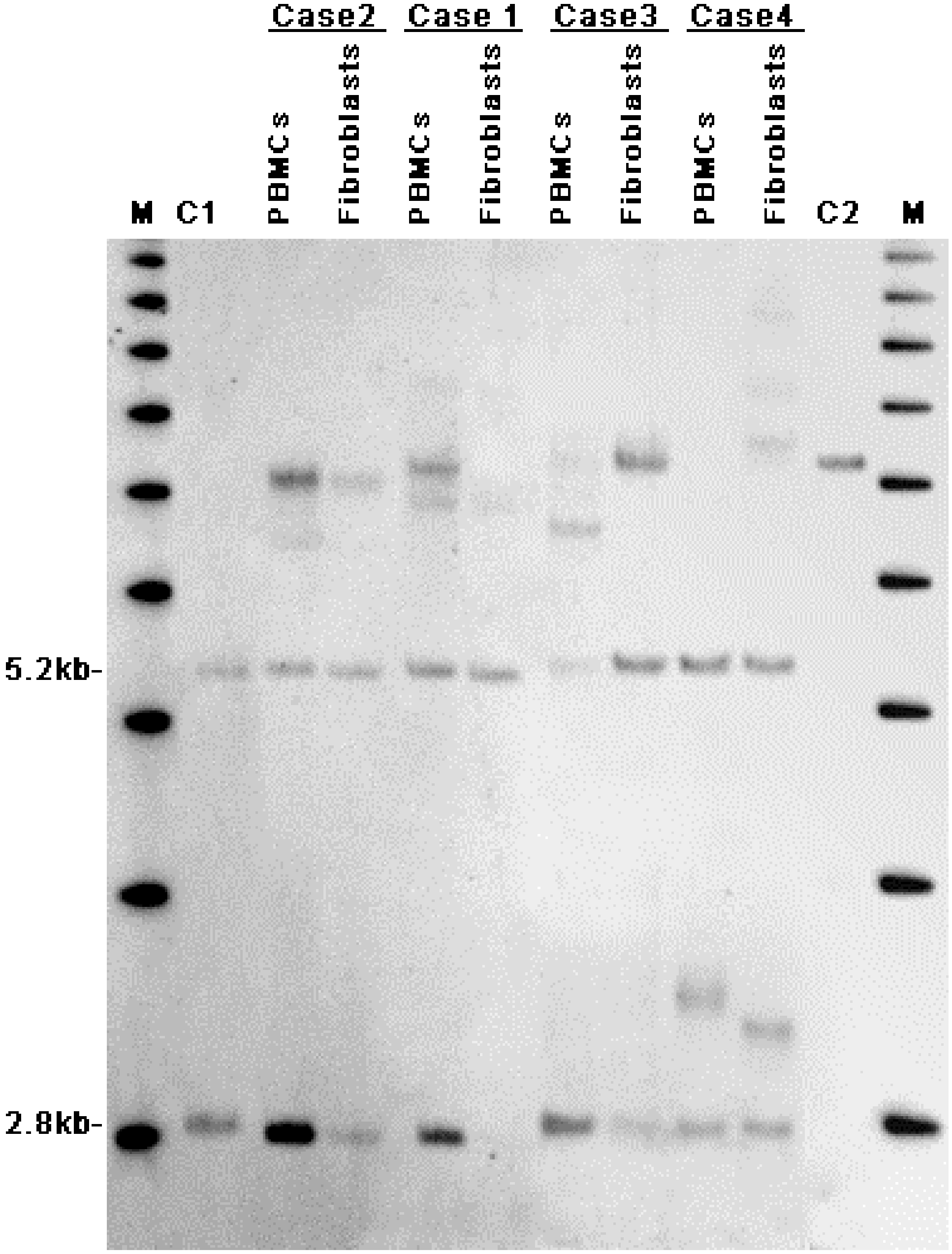

Supplement: Supplementary file 1 [file Image1.PDF]
